# Crystal structure of *cis*-bis­(μ-β-alanine-κ^2^ *O*:*O*′)bis[tri­chlorido­rhenium(III)](*Re*–*Re*) sesquihydrate

**DOI:** 10.1107/S2056989014026620

**Published:** 2015-01-01

**Authors:** Alexander A. Golichenko, Konstantin V. Domasevitch, Dina E. Kytova, Alexander V. Shtemenko

**Affiliations:** a Department of Inorganic Chemistry, Ukrainian State University of Chemical Technology, Gagarin Ave. 8, Dnipropetrovsk 49005, Ukraine; b Department of Inorganic Chemistry, National Taras Shevchenko University of Kyiv, Volodimirska Str. 64, Kyiv 01033, Ukraine

**Keywords:** crystal structure, rhenium, cluster, β-alanine, zwitterionic ammonia­carboxyl­ato complex, quadruple metal–metal bond

## Abstract

The structure of the title compound, [Re_2_Cl_6_(C_3_H_7_NO_2_)_2_]·1.5H_2_O, comprises a dinuclear complex cation [Re—Re = 2.2494 (3) Å] involving *cis*-oriented double carboxyl­ate bridges, four equatorial chloride ions and two weakly bonded chloride ligands in the axial positions at the two rhenium(III) atoms. In the crystal, two complex mol­ecules and two water mol­ecules constitute hydrogen-bonded dimers, while an extensive hydrogen-bonding network involving the groups of the zwitterionic ligand is important for generation of the framework. An additional partially occupied water molecule is disordered over two sets of sites about a symmetry centre with a site-occupancy ratio of 0.3:0.2.

## Abstract 2

A dirhenium(III) *cis*-di­carboxyl­ate complex is reported, which is representative of a small class of zwitterionic ammonia­carboxyl­ato complexes involving a quadruple metal–metal bond.

## Chemical context   

Investigations of complex compounds with multiple metal–metal bonds, which exhibit biological activity, generate great inter­est at the present stage of development of coordination chemistry (Jung & Lippard, 2007 ▸; Shtemenko *et al.*, 2013 ▸). Binuclear clusters of rhenium(III) are the classical complexes with a unique quadruple metal–metal bond (Cotton *et al.*, 2005 ▸; Golichenko & Shtemenko, 2006 ▸). In our previous studies, we have shown that these compounds can be used in medical practice as anti­tumor, anti­radical, and hepato- and nephro-protective substances with low toxicity (Dimitrov & Eastland, 1978 ▸; Shtemenko *et al.*, 2007 ▸, 2008 ▸, 2009 ▸, 2013 ▸). Labile axial ligands and equatorial chloride groups are the reactive centres not only for other substances *in vitro*, but also in inter­actions with biological macromolecules, such as proteins, DNA, and others *in vivo* (Shtemenko *et al.*, 2013 ▸). In this context, we present the synthesis and crystal structure of a new complex compound of dirhenium(III) with β-alanine as biologically active substance, which can exhibit anti­tumor activity (Shtemenko *et al.*, 2009 ▸).
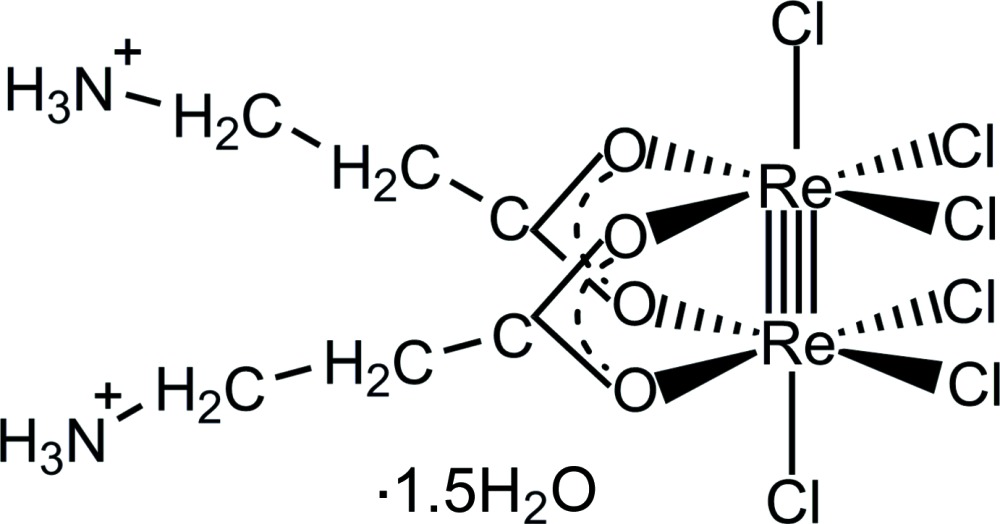


## Structural commentary   

**Figure 1:**
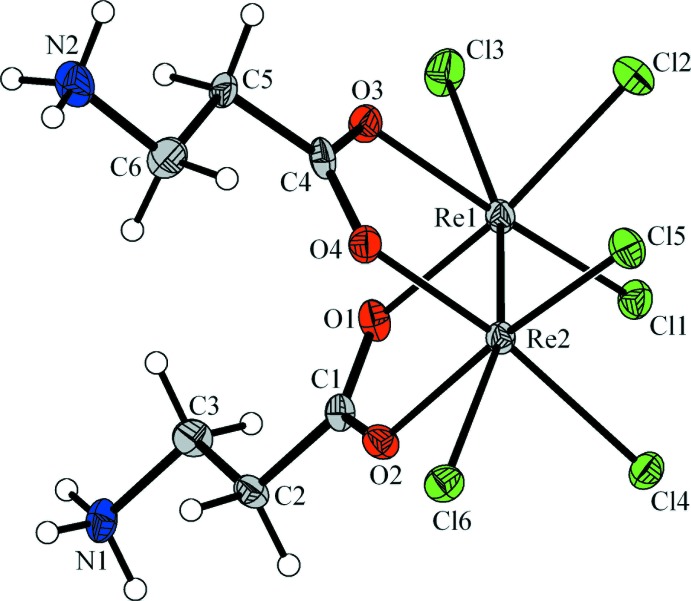
The mol­ecular structure of the title complex, with displacement ellipsoids drawn at the 40% probability level. Solvent water mol­ecules have been omitted for clarity.

It is well known that β-alanine and other amino acids are able to coordinate to a variety of transition metals (Korp *et al.*, 1981 ▸; Shtemenko *et al.*, 2009 ▸). The quadruple Re—Re bond [2.2494 (3) Å] is typical of related di­carboxyl­ato clusters (Cotton *et al.*, 2005 ▸; Shtemenko *et al.*, 2009 ▸). The octahedral coordination environment of each rhenium ion in the title compound (Fig. 1 ▸) also comprises two chloride anions and two oxygen atoms of zwitterionic alanine ligands. The distorted octa­hedral coordination of the metals is completed by weakly bonded chloride ions [Re1—Cl3 = 2.6766 (16) and Re2—Cl6 = 2.7501 (14) Å], in a *trans*-position to the Re—Re bond. This may be compared with the similar weak binding of N- or O-donors, which is characteristic of di­carboxyl­atodirhenium compounds (Bera *et al.*, 2003 ▸; Shtemenko *et al.*, 2009 ▸) and is even more appreciable for cationic tetra­carboxyl­atodirhenium species commonly accommodating a pair of chloride anions at both axial sites (Re–Cl = 2.48–2.52 Å; Shtemenko *et al.*, 2001 ▸).

## Supra­molecular features   

**Figure 2:**
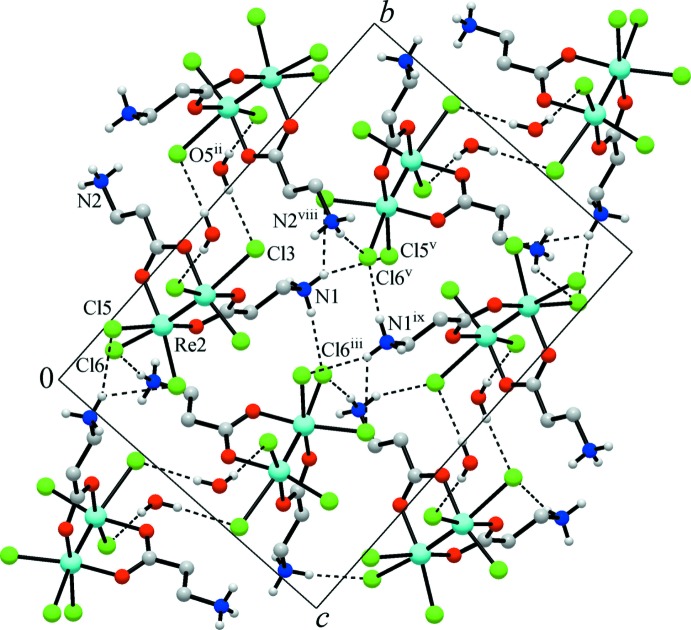
The crystal structure of the title complex viewed down the *a* axis, with the C—H hydrogens and disordered water mol­ecules omitted for clarity. Dotted lines indicate hydrogen bonds involving the OH and NH groups. Note the assembly of the hydrogen-bonded dimers constituted by two complex mol­ecules and two water mol­ecules. [Symmetry codes: (ii) −*x* + 1, −*y* + 1, −*z*; (iii) *x* + 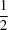, −*y* + 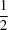, *z* + 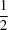; (v) −*x* + 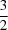, *y* + 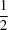, −*z* + 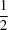; (viii) −*x*, −*y* + 1, −*z*; (ix) −*x* + 1, −*y* + 1, −*z* + 1.]

The title compound displays a three-dimensional structure dominated by weak hydrogen bonds of the O—H⋯Cl, N—H⋯Cl, C—H⋯O and C—H⋯Cl types (Table 1 ▸). The primary supra­molecular motif consists of centrosymmetric dimers (symmetry code: −*x*, −*y* + 1, −*z*) incorporating two complex moieties and two water mol­ecules (Fig. 2 ▸), with a typical hydrogen-bonding geometry [O⋯Cl = 3.342 (6) and 3.360 (6) Å], while an extensive hydrogen-bonding network involving the ammonium groups and chloride acceptors assembles the dimers into a three-dimensional framework. One of these N—H⋯Cl bonds is bifurcated and one is trifurcated (Table 1 ▸). It is worth noting that most of the N–H⋯Cl inter­actions are observed for the Cl3 and Cl6 acceptors. Such selectivity is likely predetermined by the steric accessibility and relative negative charge located at the Cl atoms, since these distal ‘axial’ chloride ligands Cl3 and Cl6 are the most underbonded and highly nucleophilic. The disordered water mol­ecules reside in the framework cages and adopt a series of short contacts, which may be attributed to weak hydrogen bonding [O⋯Cl = 3.07 (2)–3.42 (4) Å].

## Synthesis and crystallization   

1.00 g (1.25 mmol) of [β-AlaH]_2_Re_2_Cl_8_ was dissolved in 20 ml of aceto­nitrile and the solution was concentrated to half of the initial volume using a rotary evaporator. A new portion (10 ml) of the solvent was added and the solution was evaporated to half of the initial volume. This procedure was repeated five times. The dark-green crystals obtained were filtered, washed with two 5 ml portions of cold aceto­nitrile and diethyl ether and dried under vacuum at 353 K. The product (0.77 g) was recrystallized from acetone, yielding the title complex in 81% yield.

## Refinement details   

Crystal data, data collection and structure refinement details are summarized in Table 2 ▸. H atoms were refined using a riding model, with O—H = 0.85, N—H = 0.90, C—H = 0.98 Å, and with *U*_iso_(H) = 1.2*U*_eq_(C) or 1.5*U*_eq_(N,O). One of the solvate water mol­ecules is disordered over two unequal contributions, which are further disordered about an inversion centre. The refined partial occupancies for this oxygen atom (O6*A* and O6*B*) are 0.3 and 0.2, respectively. Both sites were refined anisotropically. The H atoms of the partially occupied water mol­ecule could not be located and were omitted from the final refinement.

## Supplementary Material

Crystal structure: contains datablock(s) I, New_Global_Publ_Block. DOI: 10.1107/S2056989014026620/rz5142sup1.cif

Structure factors: contains datablock(s) I. DOI: 10.1107/S2056989014026620/rz5142Isup2.hkl

CCDC reference: 1037487

Additional supporting information:  crystallographic information; 3D view; checkCIF report

## Figures and Tables

**Table 1:** Hydrogen-bond geometry (, )

*D*H*A*	*D*H	H*A*	*D* *A*	*D*H*A*
O5H1*W*Cl2^i^	0.85	2.51	3.360(6)	174
O5H2*W*Cl3^ii^	0.85	2.50	3.342(6)	174
N1H1*N*Cl6^iii^	0.90	2.32	3.202(5)	167
N1H2*N*Cl4^iv^	0.90	2.78	3.396(6)	127
N1H2*N*Cl5^iv^	0.90	2.78	3.557(5)	145
N1H2*N*Cl6^iv^	0.90	2.75	3.410(6)	131
N1H3*N*Cl3^i^	0.90	2.34	3.223(5)	167
N2H4*N*Cl6^v^	0.90	2.30	3.188(6)	172
N2H5*N*Cl2^vi^	0.90	2.84	3.575(6)	140
N2H5*N*Cl5^vi^	0.90	2.66	3.373(5)	137
N2H6*N*Cl3^ii^	0.90	2.40	3.238(7)	156
C3H3*A*O5	0.98	2.54	3.253(9)	129
C2H2*A*Cl2^i^	0.98	2.78	3.717(6)	160

Symmetry codes: (i) 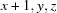; (ii) 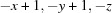; (iii) 
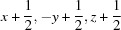
; (iv) 
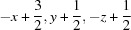
; (v) 
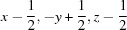
; (vi) 
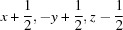
.

**Table 2:** Experimental details

Crystal data	
Chemical formula	[Re_2_Cl_6_(C_3_H_7_NO_2_)_2_]1.5H_2_O
*M*_r_	790.32
Crystal system, space group	Monoclinic, *P*2_1_/*n*
Temperature (K)	223
*a*, *b*, *c* ()	8.2884(9), 17.4526(14), 13.2715(14)
()	107.838(3)
*V* (^3^)	1827.5(3)
*Z*	4
Radiation type	Mo *K*
(mm^1^)	14.13
Crystal size (mm)	0.25 0.22 0.14

Data collection	
Diffractometer	Siemens SMART CCD area detector
Absorption correction	Multi-scan (*SADABS*; Bruker, 2008 ▸)
*T*_min_, *T*_max_	0.139, 0.267
No. of measured, independent and observed [*I* > 2(*I*)] reflections	11029, 4413, 4235
*R*_int_	0.027
(sin /)_max_ (^1^)	0.665

Refinement	
*R*[*F* ^2^ > 2(*F* ^2^)], *wR*(*F* ^2^), *S*	0.031, 0.075, 1.26
No. of reflections	4413
No. of parameters	210
H-atom treatment	H-atom parameters constrained
_max_, _min_ (e ^3^)	1.70, 1.62

Computer programs: *SMART* and *SAINT* (Bruker, 2008 ▸), *SHELXS97* and *SHELXL97* (Sheldrick, 2008 ▸), *DIAMOND* (Brandenburg, 1999 ▸) and *WinGX* (Farrugia, 2012 ▸).

## supplementary crystallographic information

### Crystal data

**Table:**

[Re_2_Cl_6_(C_3_H_7_NO_2_)_2_]·1.5H_2_O	*F*(000) = 1452
*M**_r_* = 790.32	*D*_x_ = 2.872 Mg m^−^^3^
Monoclinic, *P*2_1_/*n*	Mo *K*α radiation, λ = 0.71073 Å
*a* = 8.2884 (9) Å	Cell parameters from 11029 reflections
*b* = 17.4526 (14) Å	θ = 2.6–28.2°
*c* = 13.2715 (14) Å	µ = 14.13 mm^−^^1^
β = 107.838 (3)°	*T* = 223 K
*V* = 1827.5 (3) Å^3^	Prism, green
*Z* = 4	0.25 × 0.22 × 0.14 mm

### Data collection

**Table:**

Siemens SMART CCD area-detector diffractometer	4413 independent reflections
Radiation source: fine-focus sealed tube	4235 reflections with *I* > 2σ(*I*)
Graphite monochromator	*R*_int_ = 0.027
ω scans	θ_max_ = 28.2°, θ_min_ = 2.6°
Absorption correction: multi-scan (*SADABS*; Bruker, 2008)	*h* = −10→11
*T*_min_ = 0.139, *T*_max_ = 0.267	*k* = −23→14
11029 measured reflections	*l* = −17→17

### Refinement

**Table:**

Refinement on *F*^2^	Primary atom site location: structure-invariant direct methods
Least-squares matrix: full	Secondary atom site location: difference Fourier map
*R*[*F*^2^ > 2σ(*F*^2^)] = 0.031	Hydrogen site location: inferred from neighbouring sites
*wR*(*F*^2^) = 0.075	H-atom parameters constrained
*S* = 1.26	*w* = 1/[σ^2^(*F*_o_^2^) + (0.0324*P*)^2^ + 7.030*P*] where *P* = (*F*_o_^2^ + 2*F*_c_^2^)/3
4413 reflections	(Δ/σ)_max_ = 0.001
210 parameters	Δρ_max_ = 1.70 e Å^−^^3^
0 restraints	Δρ_min_ = −1.62 e Å^−^^3^

### Special details

**Table:**

Geometry. All e.s.d.'s (except the e.s.d. in the dihedral angle between two l.s. planes) are estimated using the full covariance matrix. The cell e.s.d.'s are taken into account individually in the estimation of e.s.d.'s in distances, angles and torsion angles; correlations between e.s.d.'s in cell parameters are only used when they are defined by crystal symmetry. An approximate (isotropic) treatment of cell e.s.d.'s is used for estimating e.s.d.'s involving l.s. planes.
Refinement. Refinement of *F*^2^ against ALL reflections. The weighted *R*-factor *wR* and goodness of fit *S* are based on *F*^2^, conventional *R*-factors *R* are based on *F*, with *F* set to zero for negative *F*^2^. The threshold expression of *F*^2^ > σ(*F*^2^) is used only for calculating *R*-factors(gt) *etc*. and is not relevant to the choice of reflections for refinement. *R*-factors based on *F*^2^ are statistically about twice as large as those based on *F*, and *R*- factors based on ALL data will be even larger.One of the solvate water molecules is disordered over center of inversion. Moreover, judging by the high anisotropy of thermal motion for this oxygen atom, two contributions of the disorder were considered and the refined partial occupancy factors were 0.20 and 0.30. Both of this contributions were refined anisotropically. However, the hydrogen atoms were not added for this disordered molecule.

### Fractional atomic coordinates and isotropic or equivalent isotropic displacement parameters (Å^2^)

**Table:**

	*x*	*y*	*z*	*U*_iso_*/*U*_eq_	Occ. (<1)
Re1	0.33803 (3)	0.336279 (13)	0.155287 (17)	0.01817 (7)	
Re2	0.46372 (3)	0.229582 (13)	0.120550 (17)	0.01690 (7)	
Cl1	0.31703 (18)	0.31409 (9)	0.32193 (11)	0.0244 (3)	
Cl2	0.05470 (17)	0.30638 (10)	0.07595 (12)	0.0279 (3)	
Cl3	0.2389 (2)	0.47984 (9)	0.17579 (14)	0.0334 (3)	
Cl4	0.48355 (18)	0.15473 (8)	0.26858 (11)	0.0234 (3)	
Cl5	0.23913 (18)	0.15472 (9)	0.02031 (11)	0.0245 (3)	
Cl6	0.66636 (18)	0.12368 (9)	0.06428 (11)	0.0241 (3)	
O1	0.5745 (5)	0.3823 (2)	0.2274 (3)	0.0223 (8)	
O2	0.6952 (5)	0.2750 (2)	0.1966 (3)	0.0218 (8)	
O3	0.3516 (5)	0.3841 (2)	0.0171 (3)	0.0235 (8)	
O4	0.4770 (5)	0.2789 (2)	−0.0169 (3)	0.0223 (8)	
N1	1.0580 (6)	0.4837 (3)	0.3589 (4)	0.0244 (10)	
H1N	1.1055	0.4574	0.4190	0.037*	
H2N	1.0596	0.5341	0.3737	0.037*	
H3N	1.1168	0.4753	0.3131	0.037*	
N2	0.5515 (7)	0.3709 (4)	−0.2897 (4)	0.0314 (12)	
H4N	0.4435	0.3778	−0.3302	0.047*	
H5N	0.6038	0.3386	−0.3225	0.047*	
H6N	0.6058	0.4162	−0.2791	0.047*	
C1	0.7059 (7)	0.3423 (3)	0.2338 (4)	0.0176 (10)	
C2	0.8792 (7)	0.3729 (3)	0.2871 (5)	0.0202 (11)	
H2A	0.9507	0.3637	0.2416	0.024*	
H2B	0.9287	0.3451	0.3535	0.024*	
C3	0.8795 (7)	0.4581 (3)	0.3108 (5)	0.0245 (12)	
H3A	0.8269	0.4866	0.2454	0.029*	
H3B	0.8143	0.4678	0.3599	0.029*	
C4	0.4226 (7)	0.3459 (4)	−0.0408 (5)	0.0209 (11)	
C5	0.4335 (7)	0.3820 (4)	−0.1409 (4)	0.0204 (11)	
H5A	0.4735	0.4349	−0.1266	0.025*	
H5B	0.3206	0.3832	−0.1932	0.025*	
C6	0.5537 (8)	0.3375 (4)	−0.1853 (5)	0.0281 (13)	
H6A	0.6688	0.3399	−0.1357	0.034*	
H6B	0.5191	0.2836	−0.1945	0.034*	
O5	0.7596 (7)	0.4427 (4)	0.0542 (4)	0.0549 (16)	
H1W	0.8330	0.4072	0.0644	0.082*	
H2W	0.7539	0.4649	−0.0038	0.082*	
O6B	0.464 (7)	0.497 (3)	0.458 (3)	0.072 (15)	0.20
O6A	0.472 (3)	0.4887 (13)	0.4049 (19)	0.051 (6)	0.30

### Atomic displacement parameters (Å^2^)

**Table:**

	*U*^11^	*U*^22^	*U*^33^	*U*^12^	*U*^13^	*U*^23^
Re1	0.01700 (11)	0.01642 (12)	0.02398 (12)	0.00109 (8)	0.01056 (8)	0.00113 (8)
Re2	0.01678 (11)	0.01375 (12)	0.02137 (11)	−0.00057 (8)	0.00761 (8)	0.00023 (8)
Cl1	0.0256 (6)	0.0265 (7)	0.0241 (6)	−0.0002 (6)	0.0121 (5)	0.0022 (6)
Cl2	0.0179 (6)	0.0335 (8)	0.0326 (7)	0.0021 (6)	0.0080 (5)	−0.0022 (6)
Cl3	0.0406 (8)	0.0206 (7)	0.0484 (9)	0.0069 (7)	0.0273 (7)	0.0041 (7)
Cl4	0.0268 (7)	0.0198 (7)	0.0249 (6)	0.0005 (5)	0.0097 (5)	0.0040 (5)
Cl5	0.0212 (6)	0.0259 (7)	0.0257 (6)	−0.0053 (5)	0.0059 (5)	−0.0044 (6)
Cl6	0.0268 (6)	0.0206 (7)	0.0254 (6)	0.0031 (6)	0.0089 (5)	−0.0005 (5)
O1	0.0208 (18)	0.020 (2)	0.029 (2)	−0.0041 (16)	0.0123 (16)	−0.0047 (17)
O2	0.0180 (18)	0.018 (2)	0.028 (2)	0.0003 (16)	0.0055 (16)	−0.0019 (16)
O3	0.0242 (19)	0.021 (2)	0.030 (2)	0.0042 (17)	0.0158 (17)	0.0039 (17)
O4	0.0220 (19)	0.021 (2)	0.026 (2)	−0.0003 (17)	0.0108 (16)	0.0018 (17)
N1	0.029 (2)	0.020 (3)	0.026 (2)	−0.009 (2)	0.010 (2)	−0.001 (2)
N2	0.028 (3)	0.044 (4)	0.028 (3)	0.001 (3)	0.017 (2)	0.000 (2)
C1	0.020 (2)	0.016 (3)	0.019 (2)	−0.003 (2)	0.009 (2)	−0.003 (2)
C2	0.015 (2)	0.019 (3)	0.026 (3)	0.001 (2)	0.006 (2)	−0.002 (2)
C3	0.024 (3)	0.013 (3)	0.035 (3)	0.001 (2)	0.007 (2)	−0.004 (2)
C4	0.019 (2)	0.023 (3)	0.026 (3)	−0.004 (2)	0.015 (2)	−0.002 (2)
C5	0.021 (2)	0.023 (3)	0.022 (3)	0.002 (2)	0.013 (2)	0.007 (2)
C6	0.033 (3)	0.026 (3)	0.030 (3)	0.006 (3)	0.016 (3)	0.004 (3)
O5	0.051 (3)	0.074 (5)	0.044 (3)	0.020 (3)	0.023 (3)	0.025 (3)
O6B	0.10 (4)	0.05 (2)	0.08 (3)	0.03 (2)	0.05 (4)	−0.01 (3)
O6A	0.064 (15)	0.023 (10)	0.057 (14)	0.003 (10)	0.003 (12)	0.003 (11)

### Geometric parameters (Å, º)

**Table:**

Re1—O3	2.049 (4)	N2—C6	1.498 (8)
Re1—O1	2.063 (4)	N2—H4N	0.9000
Re1—Re2	2.2494 (3)	N2—H5N	0.9000
Re1—Cl1	2.3037 (14)	N2—H6N	0.9000
Re1—Cl2	2.3197 (14)	C1—C2	1.492 (7)
Re1—Cl3	2.6766 (16)	C2—C3	1.519 (8)
Re2—O2	2.035 (4)	C2—H2A	0.9800
Re2—O4	2.050 (4)	C2—H2B	0.9800
Re2—Cl4	2.3227 (14)	C3—H3A	0.9800
Re2—Cl5	2.3291 (14)	C3—H3B	0.9800
Re2—Cl6	2.7501 (14)	C4—C5	1.498 (7)
O1—C1	1.273 (7)	C5—C6	1.518 (8)
O2—C1	1.267 (7)	C5—H5A	0.9800
O3—C4	1.288 (7)	C5—H5B	0.9800
O4—C4	1.258 (7)	C6—H6A	0.9800
N1—C3	1.490 (7)	C6—H6B	0.9800
N1—H1N	0.9000	O5—H1W	0.8500
N1—H2N	0.9000	O5—H2W	0.8500
N1—H3N	0.9000		
			
O3—Re1—O1	87.24 (17)	H2N—N1—H3N	109.5
O3—Re1—Re2	89.88 (12)	C6—N2—H4N	109.5
O1—Re1—Re2	89.05 (12)	C6—N2—H5N	109.5
O3—Re1—Cl1	165.62 (13)	H4N—N2—H5N	109.5
O1—Re1—Cl1	87.73 (12)	C6—N2—H6N	109.5
Re2—Re1—Cl1	103.50 (4)	H4N—N2—H6N	109.5
O3—Re1—Cl2	90.63 (13)	H5N—N2—H6N	109.5
O1—Re1—Cl2	169.84 (13)	O2—C1—O1	121.7 (5)
Re2—Re1—Cl2	100.88 (4)	O2—C1—C2	117.3 (5)
Cl1—Re1—Cl2	91.96 (5)	O1—C1—C2	121.0 (5)
O3—Re1—Cl3	79.19 (12)	C1—C2—C3	112.9 (5)
O1—Re1—Cl3	82.31 (12)	C1—C2—H2A	109.0
Re2—Re1—Cl3	166.33 (4)	C3—C2—H2A	109.0
Cl1—Re1—Cl3	86.79 (5)	C1—C2—H2B	109.0
Cl2—Re1—Cl3	87.54 (6)	C3—C2—H2B	109.0
O2—Re2—O4	88.74 (17)	H2A—C2—H2B	107.8
O2—Re2—Re1	90.07 (11)	N1—C3—C2	108.8 (5)
O4—Re2—Re1	89.38 (12)	N1—C3—H3A	109.9
O2—Re2—Cl4	89.33 (12)	C2—C3—H3A	109.9
O4—Re2—Cl4	168.37 (12)	N1—C3—H3B	109.9
Re1—Re2—Cl4	102.09 (4)	C2—C3—H3B	109.9
O2—Re2—Cl5	165.67 (12)	H3A—C3—H3B	108.3
O4—Re2—Cl5	88.78 (12)	O4—C4—O3	121.6 (5)
Re1—Re2—Cl5	104.01 (4)	O4—C4—C5	120.0 (5)
Cl4—Re2—Cl5	90.27 (5)	O3—C4—C5	118.3 (5)
O2—Re2—Cl6	80.51 (12)	C4—C5—C6	110.8 (5)
O4—Re2—Cl6	80.62 (12)	C4—C5—H5A	109.5
Re1—Re2—Cl6	166.35 (3)	C6—C5—H5A	109.5
Cl4—Re2—Cl6	87.75 (5)	C4—C5—H5B	109.5
Cl5—Re2—Cl6	85.16 (5)	C6—C5—H5B	109.5
C1—O1—Re1	119.3 (4)	H5A—C5—H5B	108.1
C1—O2—Re2	119.8 (4)	N2—C6—C5	109.7 (5)
C4—O3—Re1	118.9 (4)	N2—C6—H6A	109.7
C4—O4—Re2	120.1 (4)	C5—C6—H6A	109.7
C3—N1—H1N	109.5	N2—C6—H6B	109.7
C3—N1—H2N	109.5	C5—C6—H6B	109.7
H1N—N1—H2N	109.5	H6A—C6—H6B	108.2
C3—N1—H3N	109.5	H1W—O5—H2W	108.4
H1N—N1—H3N	109.5		
			
O3—Re1—Re2—O2	89.01 (17)	O4—Re2—O2—C1	86.9 (4)
O1—Re1—Re2—O2	1.77 (16)	Re1—Re2—O2—C1	−2.5 (4)
Cl1—Re1—Re2—O2	−85.66 (12)	Cl4—Re2—O2—C1	−104.6 (4)
Cl2—Re1—Re2—O2	179.63 (13)	Cl5—Re2—O2—C1	166.9 (4)
Cl3—Re1—Re2—O2	52.4 (2)	Cl6—Re2—O2—C1	167.6 (4)
O3—Re1—Re2—O4	0.28 (16)	O1—Re1—O3—C4	87.3 (4)
O1—Re1—Re2—O4	−86.96 (16)	Re2—Re1—O3—C4	−1.7 (4)
Cl1—Re1—Re2—O4	−174.40 (12)	Cl1—Re1—O3—C4	157.0 (4)
Cl2—Re1—Re2—O4	90.90 (12)	Cl2—Re1—O3—C4	−102.6 (4)
Cl3—Re1—Re2—O4	−36.4 (2)	Cl3—Re1—O3—C4	170.0 (4)
O3—Re1—Re2—Cl4	178.34 (13)	O2—Re2—O4—C4	−89.0 (4)
O1—Re1—Re2—Cl4	91.10 (12)	Re1—Re2—O4—C4	1.1 (4)
Cl1—Re1—Re2—Cl4	3.67 (5)	Cl4—Re2—O4—C4	−169.5 (5)
Cl2—Re1—Re2—Cl4	−91.04 (6)	Cl5—Re2—O4—C4	105.2 (4)
Cl3—Re1—Re2—Cl4	141.70 (18)	Cl6—Re2—O4—C4	−169.6 (4)
O3—Re1—Re2—Cl5	−88.31 (13)	Re2—O2—C1—O1	1.8 (7)
O1—Re1—Re2—Cl5	−175.54 (12)	Re2—O2—C1—C2	−179.0 (4)
Cl1—Re1—Re2—Cl5	97.02 (5)	Re1—O1—C1—O2	0.3 (7)
Cl2—Re1—Re2—Cl5	2.31 (6)	Re1—O1—C1—C2	−178.9 (4)
Cl3—Re1—Re2—Cl5	−124.95 (18)	O2—C1—C2—C3	169.6 (5)
O3—Re1—Re2—Cl6	42.93 (18)	O1—C1—C2—C3	−11.2 (8)
O1—Re1—Re2—Cl6	−44.31 (18)	C1—C2—C3—N1	−177.3 (5)
Cl1—Re1—Re2—Cl6	−131.75 (14)	Re2—O4—C4—O3	−2.8 (7)
Cl2—Re1—Re2—Cl6	133.55 (14)	Re2—O4—C4—C5	179.6 (4)
Cl3—Re1—Re2—Cl6	6.3 (2)	Re1—O3—C4—O4	3.1 (7)
O3—Re1—O1—C1	−91.7 (4)	Re1—O3—C4—C5	−179.3 (4)
Re2—Re1—O1—C1	−1.8 (4)	O4—C4—C5—C6	−15.0 (8)
Cl1—Re1—O1—C1	101.8 (4)	O3—C4—C5—C6	167.2 (5)
Cl2—Re1—O1—C1	−169.8 (5)	C4—C5—C6—N2	175.4 (5)
Cl3—Re1—O1—C1	−171.2 (4)		

### Hydrogen-bond geometry (Å, º)

**Table:**

*D*—H···*A*	*D*—H	H···*A*	*D*···*A*	*D*—H···*A*
O5—H1*W*···Cl2^i^	0.85	2.51	3.360 (6)	174
O5—H2*W*···Cl3^ii^	0.85	2.50	3.342 (6)	174
N1—H1*N*···Cl6^iii^	0.90	2.32	3.202 (5)	167
N1—H2*N*···Cl4^iv^	0.90	2.78	3.396 (6)	127
N1—H2*N*···Cl5^iv^	0.90	2.78	3.557 (5)	145
N1—H2*N*···Cl6^iv^	0.90	2.75	3.410 (6)	131
N1—H3*N*···Cl3^i^	0.90	2.34	3.223 (5)	167
N2—H4*N*···Cl6^v^	0.90	2.30	3.188 (6)	172
N2—H5*N*···Cl2^vi^	0.90	2.84	3.575 (6)	140
N2—H5*N*···Cl5^vi^	0.90	2.66	3.373 (5)	137
N2—H6*N*···Cl3^ii^	0.90	2.40	3.238 (7)	156
C3—H3*A*···O5	0.98	2.54	3.253 (9)	129
C2—H2*A*···Cl2^i^	0.98	2.78	3.717 (6)	160

Symmetry codes: (i) *x*+1, *y*, *z*; (ii) −*x*+1, −*y*+1, −*z*; (iii) *x*+1/2, −*y*+1/2, *z*+1/2; (iv) −*x*+3/2, *y*+1/2, −*z*+1/2; (v) *x*−1/2, −*y*+1/2, *z*−1/2; (vi) *x*+1/2, −*y*+1/2, *z*−1/2.
